# The rationale and emergence of electroconductive biomaterial scaffolds in cardiac tissue engineering

**DOI:** 10.1063/1.5116579

**Published:** 2019-10-15

**Authors:** Matteo Solazzo, Fergal J. O'Brien, Valeria Nicolosi, Michael G. Monaghan

**Affiliations:** 1 Department of Mechanical and Manufacturing Engineering, Trinity College Dublin, Dublin 2, Ireland; 2 Trinity Centre for BioEngineering, Trinity College Dublin, Dublin 2, Ireland; 3 Advance Materials and BioEngineering Research (AMBER) Centre at Trinity College Dublin and the Royal College of Surgeons in Ireland, Dublin 2, Ireland; 4 Tissue Engineering Research Group (TERG), Dublin 2, Ireland; 5 Department of Anatomy, Royal College of Surgeons in Ireland (RCSI), Dublin 2, Ireland; 6 School of Chemistry, Trinity College Dublin, Dublin 2, Ireland; 7 Centre for Research on Adaptive Nanostructures and Nanodevices (CRANN), Trinity College Dublin, Dublin 2, Ireland

## Abstract

The human heart possesses minimal regenerative potential, which can often lead to chronic heart failure following myocardial infarction. Despite the successes of assistive support devices and pharmacological therapies, only a whole heart transplantation can sufficiently address heart failure. Engineered scaffolds, implantable patches, and injectable hydrogels are among the most promising solutions to restore cardiac function and coax regeneration; however, current biomaterials have yet to achieve ideal tissue regeneration and adequate integration due a mismatch of material physicochemical properties. Conductive fillers such as graphene, carbon nanotubes, metallic nanoparticles, and MXenes and conjugated polymers such as polyaniline, polypyrrole, and poly(3,4-ethylendioxythiophene) can possibly achieve optimal electrical conductivities for cardiac applications with appropriate suitability for tissue engineering approaches. Many studies have focused on the use of these materials in multiple fields, with promising effects on the regeneration of electrically active biological tissues such as orthopedic, neural, and cardiac tissue. In this review, we critically discuss the role of heart electrophysiology and the rationale toward the use of electroconductive biomaterials for cardiac tissue engineering. We present the emerging applications of these smart materials to create supportive platforms and discuss the crucial role that electrical stimulation has been shown to exert in maturation of cardiac progenitor cells.

## I. INTRODUCTION

Cardiac muscle relies on an intricate coordination of action potentials and calcium signal propagation in order to exert synchronous beating to pump blood around our bodies. This coordination is facilitated by membrane potential depolarization, a pacemaker conduction system, and specific intracellular communication networks.^1^ Dysregulation of these processes can occur due to cardiac arrhythmic conditions, such as sinus node dysfunction or atrioventricular (AV) block, and, depending on the severity of the pathology, a pacemaker device may be implemented to restore cardiac synchronicity.^2^

Quite often, such coordination becomes interrupted due to ischemic death of myocardial muscle stemming from the advent of atherosclerosis and myocardial infarction (MI), more commonly known as a “heart attack.”^3–6^ This ischemic insult to myocardial muscle often results in the formation of a fibrotic scar which, although lends some compensatory role to replace the necrotic myocardial core, is relatively inert to the electric signaling of the heart acting as an insulating tissue that isolates remote cardiomyocytes (CMs) and impedes communication of healthy tissue. Electrophysiological characterization of the noncontractile infarcted region has identified two interesting phenomena: the presence of tortuous propagation pathways that lend some degree (although much reduced) of conductivity to the fibrotic scar tissue and the induction of ventricular tachyarrhythmia due to strands of viable cardiomyocytes that permeate through the scar volume.^7^ Heterotypic coupling between fibroblasts and myocytes plays a significant role in the development of pathological conditions.^8^ Fibroblasts can also transmit action potentials between isolated CMs, propagate electrical waves through the scar, and, at high densities, prevent arrhythmias.^7^ Additionally, further hindrance to the transmission of action potentials within the heart is the occurrence of morbid hypertrophic compensatory mechanisms that have been shown to possibly lead to arrhythmogenesis.^9^

The myocardial milieu has minimal regenerative potential with an estimated cardiomyocyte turnover of 1% per year at the age of 25 and 0.45% at the age of 75.^10,11^ Because of this, MI can lead to cardiac hypertrophy, myocyte slippage, arrhythmia, tachycardia, and even complete heart failure. Current pharmaceutical treatments strive to alleviate further deterioration of cardiac function by administration of β-blockers, aspirin, thrombolytics, antiplatelet agents, and angiotensin-converting enzyme inhibitors yet do not stimulate regeneration.^11,12^ Moreover, despite the rapidly rising field of biomedical instrumentation and life assisting devices for heart support such as ventricle assisting devices and intra-aortic balloon pumps,^13^ these solutions can only be considered as bridge therapies to an “ideal” yet limited treatment for heart failure: whole heart transplantation. It is estimated that only 10% of the patients requiring whole heart transplantation will benefit from such in their lifetime.^14^

Tissue engineering strategies are being increasingly focused upon to deliver the next generation of treatments for ischemic myocardium, with the main aim to recapitulate the cardiac microenvironment through mechanical, topographical, and extracellular matrix (ECM)-mediated cues.

Moreover, in parallel to the established tissue regenerative medicine paradigm, tissue engineering is moving toward the development of *in vitro* disease models rapidly, which possess direct translational outcomes and less strict regulatory issues.^15^ Indeed, organoids—three dimensional constructs formed by the aggregation of cells *in vitro*—derived from a patient's own cells can facilitate identification of suitable pharmacological therapy for specific cohorts of patients, obviating adverse drug reactions and opening the discovery of new treatments.^15,16^

Efforts for *in vivo* tissue repair have been multifactorial and use a number of different avenues, which can consist of biomaterial scaffolds, cell therapies,^17,18^ gene therapy,^19^ and localized drug delivery or their combination. Local application of biomaterials has been postulated as a beneficial treatment with collateral support and mechanical strengthening being one of the mechanisms hypothesized to stem from this treatment.^20^ Typical biomaterials can be relatively inert in nature, composed of either synthetic or natural polymers or a combination of both, and exist in forms of injectable hydrogels,^21^ geometrically defined scaffolds,^22^ particulates,^23^ or as substrate coatings.^24^ Such materials can possess predefined mechanical properties with adequate biocompatibility and often have been reported to improve^25^ or maintain myocardial function^26^ but essentially exist as inert depots and at the most adding some mechanical support to the compromised myocardium. Such materials have evolved though to possess additional complexity with the incorporation of cells,^27^ drugs,^28^ and gene therapy.^29^

From a design point of view, one must appreciate that the myocardium exists as a contractile, active tissue with continuous cycles of ionic polarization and depolarization which can adapt to demands in corporeal oxygen demand, and therefore, a tissue engineering approach must meet these design criteria. First, the material of choice must exhibit suitable biocompatibility promoting interaction with cells and avoiding both short- and long-term toxicity.^30^ Specific criteria related to the scaffold architecture at the multiscale level include high porosity and adequate pore size to allow cell infiltration for both *in vitro* derived platforms and *in vivo* repopulation of the scaffold;^31^ moreover, pore geometry has been shown to play a crucial role directing cardiac tissue maturation and assembly, with aligned topography promoting intramyocyte communication.^32,33^ Once these requisites are accomplished, one should also consider host physicochemical properties to enhance cell engagement such as substrate stiffness and flexibility.^34^ Another physicochemical property gathering significant momentum in recent years is that of the conductivity of biomaterials matching the bioconductance of the native myocardium.^35^ Meeting such a criterion could restore myocardial/chamber signaling and re-establish efficient synchronous beating to hinder further myocardial aggravations as deterioration, slipping, or hypertrophy. Despite electrical activity being a key feature of several functions and organs in the human body,^36^ to date, most materials adopted for tissue engineering have not been designed with this feature in mind. Electrically conductive biomaterials investigated in the field can be categorized as either extrinsically conductive materials—predominantly fashioned by the incorporation of conductive fillers into an insulating material matrix—or intrinsically conductive polymers. Despite their different origin and mechanisms of conductivity, both these families can be applied in cardiac tissue engineering, due to their ease of manipulation and processing in combination with other materials, metallike conductivity, and biocompatibility.^37^ Their application in tissue engineering is rapidly expanding; still much has to be determined with regard to their long-term impact and potency in regenerating tissue *in vivo*. The complex electrical pathways of the myocardium must be fully appreciated and understood with a goal to achieving biomaterial chemistries, morphologies, and optimal tissue/material interfaces to exert a maximum benefit. This review is a discussion of this burgeoning field in adopting electroconductive materials to treat MI by their application and in achieving cardiac organoids to study cardiac disease.

## II. CARDIAC ELECTROPHYSIOLOGY

To begin our understanding, we consider first the myocardium, the involuntary striated muscular tissue occupying the inner mass of the heart wall and the major proponent of contractility, present as a framework of parallel myofibers. These fibers are precisely oriented across the myocardium, conferring the organ's characteristic twist during the contraction cycle^38,39^ and are composed of a group of contractile muscle cells—cardiomyocytes (CMs)—and held together by strands of connective tissue.^8^ Along with these CMs, supporting resident cells are present such as endothelial cells, smooth muscle cells, macrophages, and cardiac fibroblasts which play a key role in the remodeling of the heart during development and pathological conditions.^8^ Surrounding these cells and making up 5% of myocardial dry weight, the cardiac-specific extracellular matrix (ECM) provides architectural support to the myofibers and plays a crucial role in the mechanotransduction of surrounding cells, causing changes in the morphology and deposition of structural and functional proteins.^40^ The contractile machine of the heart is strictly dependent on the function of the electrical propagation pathway that consists in pacemaker cells atrial, atrioventricular, and Purkinje cells. These populations are present in the heart, and they are specifically associated with initiating and conducting impulses to the ventricular contractile cells.^38^

### A. Cardiac electroconductivity

For the heart to contract rhythmically, each sarcomere, the functional and structural unit of the cardiac muscle, within a particular muscle fiber must shorten coincidently.^41^ Cardiac electrical activity is initiated in the sinoatrial (SA) node, a discrete mass of specialized cells located in the right atrium. This electrical stimulus is generated at about 60–100 times per minute at regular intervals.^41^ When the action potential is at −65 mV, diastolic depolarization begins, and at −45 mV, the nodal action potential is triggered. As the electrical signal propagates along the atria toward the atrioventricular (AV) node, it stimulates the atria to contract. Continuing to the bundle of *His*, this signal reaches the Purkinje fibers that extend and propagate throughout the myocardium which allows for each cell within the myocardium to experience this action potential.^1^

Instigators of myocardial contraction, actin and myosin, become activated in the presence of ions, specifically of which are calcium ions that are responsible for the cross-bridge binding, the process through tropomyosin molecules shift in the presence of calcium ions to expose the myosin binding sites on the actin, and enabling the relative sliding of the two molecules.^38^ Structures termed gap junctions ensure that the action potential can rapidly spread throughout the muscle fiber network to allow the myocardium to function as a single unit,^7^ with conductivity values of approximately 0.48 S/m in the atria,^36^ and 0.3–0.6 S/m within the ventricles.^42,43^ Two other cell junction types, adherens and desmosomes, add further to the mechanical functions by ensuring that the mechanical forces are transmitted throughout the myocardium.^44,45^

In the ischemic heart, necrosed CMs are replaced by laying down a noncontractile crosslinked collagen rich scar that hinder contraction propagation.^7^ This leads to a disruption in this cellular connectivity, and altered ion-channel activity occurs and eventually leads to contractile disfunctions.^7,46^

## III. ELECTROCONDUCTIVE BIOMATERIALS

Recognizing this important and intricate role of electrical signaling in the native myocardium, its dysregulation during disease, and recent bounds in research appreciating bioelectrical signaling, electroconductive biomaterials have emerged as a new class of building blocks in tissue engineering in a wide range of applications extending from neural,^47^ musculoskeletal^48^ to cardiac.^35^ Scaffolds and conduits for regenerative medicine have yet to fulfill several requirements to successfully support and drive cell behavior and to achieve mature and functional tissue formation. Biomaterials used to fabricate such structures not only need to mimic physiological electroconductivity values but also possess other desired criteria such as biocompatibility and adequate degradation kinetics. These factors—together with the intrinsic chemical and physical properties of the chosen compound—are all important factors that dictate cytotoxicity and, therefore, the overall outcome. A large portion of studies with these materials has involved some limited *in vitro* work with some direct investigation in neural and orthopedic applications. However, cardiac tissue engineering applications have recently garnered focus with much success.

### A. Extrinsically conductive materials

Extrinsically conductive materials are generally considered compounds becoming electrically conductive due to the combination of an insulating material with a conductive filler, defining as a percolation threshold the minimum content of filler necessary to achieve the transition to the conductive state.^49^ Despite concerns on their long-term effects as implants in the body, these materials are increasingly pursued due to the ease at which they can be processed and manipulated and incorporated with therapeutic natural polymers (ECM) and capability to be manufactured in large scale processes.

#### 1. Carbon nanotubes (CNTs)

The hallmark synthesis of carbon nanotubes (CNTs) in 1991 paved the way for bounding advancement in nanotechnology.^50^ CNTs are sheets of graphite rolled into cylindrical tubes consisting of diameters in the range of 0.4–2 nm with lengths much longer ranging from hundreds of nanometers to micrometers.^51^ Such varying aspect ratios can be manipulated in tissue engineering design to mimic the intrinsic anisotropic properties of some native tissues, such as bones or muscle fibers. They can further be divided into single-walled CNTs (SWCNTs) and multiwalled CNTs (MWCNTs) depending on their geometry. Their superior properties, such as a 11–200 GPa tensile strength,^52^ Young's modulus of 0.27–1.34 TPa,^50,53^ electrical conductivities from 1 × 10^4^ S/cm^2^, and thermal conductivities at 5000 W/m K,^54,55^ are known to improve the mechanical and chemical properties of biomaterials and polymers. The benefits of introducing these particles have been evident in tissue engineering since the early 2000s, notably in the fields of neural, bone, and cardiac regeneration with enhancement of tissue maturation.^56^ The presence of CNTs has been suggested to elicit an antioxidant response with a free radical scavenger mechanism, which has been tested via infusion of doxorubicin, and attributed to adduct formation and neutralization through electron transfer.^35^

#### 2. Graphene

Pristine graphene is collectively defined as one-atom-thick flat sheets of carbon initially obtained via a simple “Scotch-tape” method to peel atomically thin layers^57^ and later by epitaxial chemical vapor deposition (CVD).^58^ Because of its unique structure, pristine graphene has been considered the thinnest and strongest material ever reported, manifesting superior electrical and optical conductive properties.^59^ Graphene can be configured as graphene oxide (GO), a less pure version but more suitable for large-scale manufacturing.^60^ The hydrophilic structure of GO is usually achieved via graphene liquid-phase exfoliation of a flaked graphite precursor, which yields an impure structure where epoxides, alcohols, ketone carbonyls, and carboxylic groups can contaminate the contiguous aromatic lattice.^61^ GO possesses a nonconductive state and a chemical reduction process is necessary to achieve reduced GO (rGO), via exposure of hydrazine vapor merged with low-temperature annealing treatment.^62^ Overall, graphene and its derivatives possess exceptional thermal, electrical, and mechanical properties, gaining an increasing amount of attention in the past decade.^63^ Since the first use of GO as a nanocarrier for drug delivery,^64^ several applications have been proposed in the biomedical field, specifically in tissue engineering for bone,^65^ nerve,^66^ and cardiac regeneration.^67^ Graphene exhibits lower cytotoxicity compared to CNTs, while the duration of the reduction process has revealed to be pivotal for cell survival, with the best results in cell response with 90 min of reduction treatment.^68^

#### 3. Metallic nanoparticles

Silver (Ag), gold (Au), and their combined alloy (AgAu) have been among the first materials ever used in the history of medicine. Gold has been used in medicine since 2500 B.C., and in its metallic form, it is unreactive and insoluble.^69^ Silver inhibits enzymatic systems of the respiratory chain and alters DNA synthesis of bacteria via superficial contact, showing an outstanding antimicrobial activity also as nanoparticles (NPs)^70^ and thanks to the ability to address the multidrug resistance of bacteria, it is considered a valuable alternative to antibiotics.^71^ NPs are defined as elements of size ranging between 1 and 100 nm;^72^ they can be manufactured either via a “top-down” approach from a macroscale material to a nanometric scale adopting mechanical techniques such as milling,^73^ or via a “bottom-up” strategy starting from an atomic/molecular level and scaling up with chemical and physical processes like aerosol or precipitation processes.^74^ Although palladium^75^ and magnetic iron oxide^76^ are also used to manufacture NPs, to date, AgNPs, AuNPs, and AgAuNPs are the most common choices in biomedical applications with potential use as nanoscale drug carriers and anticancer treatments.^77,78^ The geometry of NPs is crucial for cell uptake, and it is reported that AuNPs with a diameter of 50 nm and an aspect ratio of 1:1 are absorbed most into mammalian cells.^79^

In the field of tissue engineering, incorporation of AgNPs and AuNPs into hydrogels has been the most common approach to generate a functionalized conductive biomaterial with NPs.^80,81^ The presence of AgNPs and AuNPs is generally well tolerated by a variety of cells *in vitro.*^82–84^ *In vivo* studies, investigating the regeneration soft tissue and bone reports an anti-inflammatory action of NP loaded hydrogels in collagen, hyaluronic acid-hydrogels, and GelMA.^85^ Several factors may have contributed to all the beneficial effects of NPs on cells and tissues reported by their applications *in vitro* and *in vivo*, such as their absorption into cell cytoplasm and nuclei,^86^ the increase in stiffness and electrical conductivity they infer,^87^ and also the modifications in nanometric topography and roughness.^88^ However, it is important to consider the potential toxic effect of introducing NPs into the body,^89^ as it has been reported when whereby a size dependent toxicity has been demonstrated when delivering AgNPs to the lungs.^90^ Comparing the effects of both AgNPs and AuNPs, it is found that Ag possesses a stronger antimicrobial activity, but its dose must be tightly controlled as it can show much higher cytotoxicity, especially for high concentrations.^84^

#### 4. MXenes

As the rising star in the 2D family, transition metal carbides and nitrides, known as MXenes, have emerged and rapidly drawn intensive research attention.^91,92^ MXenes were developed by Barsoum and co‐workers. The Mn + 1Xn layer (named as MXene) was fabricated by the selective extraction of the A‐element from layered ternary carbides of Mn + 1AXn phases (n = 1–3), where M is an early transition metal, A is an A group element, and X is C or N.^91–94^ MXenes typically have three different formulas: M2X, M3X2, and M4X3. Being the most widely investigated MXene type, titanium carbide (Ti3C2Tx, Tx stands for various surface functionalities such as –OH, –O, and/or –F, and n = 1, 2, or 3^95,96^) exhibits a metallic conductivity and excellent capacitive charge storage behavior.^97–99^

Since the discovery of Ti3C2 in 2011, the family of transition metal carbides, carbonitrides, and nitrides, collectively referred to as MXenes, has quickly expanded in many areas. By selective etching of A-element layers from the MAX precursor in aqueous fluoride-containing acidic solutions, such as hydrofluoric acid, HF, or *in situ* formed HF from lithium fluoride and hydrochloric acid, LiF + HCl, or ammonium hydrogen bifluoride (NH_4_HF_2_), multilayered (m-)MXene is thus obtained. The abundant surface functional groups impart hydrophilicity to MXenes. When m-MXene is delaminated into monolayered or few-layered nanosheets (d-MXene), a stable aqueous solution can be thus obtained, due to the electrostatic force on the negatively charged MXene nanosheets. This allows a facile and environmentally friendly processing of the MXene solution into any items, such as composites, coatings, and devices. Despite the presence of terminal surface groups, MXenes, especially the most intensively studied titanium carbide MXene (Ti_3_C_2_T_x_), showcase a metallic conductivity as high as 9880 S cm^−1^ (Ref. 100). Compared to other metallic mesh and carbon nanomaterials, Ti_3_C_2_T_x_ MXene nanosheets have a series of advantages including high flexibility, ease of dispersion in water, and biocompatibility. To date, these 2D multifunctional MXenes and their composites have, for example, been developed for theranostic applications including typical phototherapy of photothermal therapy (PTT), photothermal/photodynamic/chemosynergistic therapy, diagnostic imaging, antimicrobial, and biosensing.^98,101–106^

Recently, MXene quantum dots have been investigated for their biocompatibility and immunomodulatory potential, showing that they can exert an anti‐inflammatory effect by decreasing the human T‐cell‐dependent inflammation in a cytocompatible fashion. Moreover, the high biocompatibility of these particles and the possibility to incorporate them into hydrogels highlight their potentiality for tissue engineering,^107^ which has yet to be investigated.

### B. Intrinsically conductive polymers

**FIG. 1.**
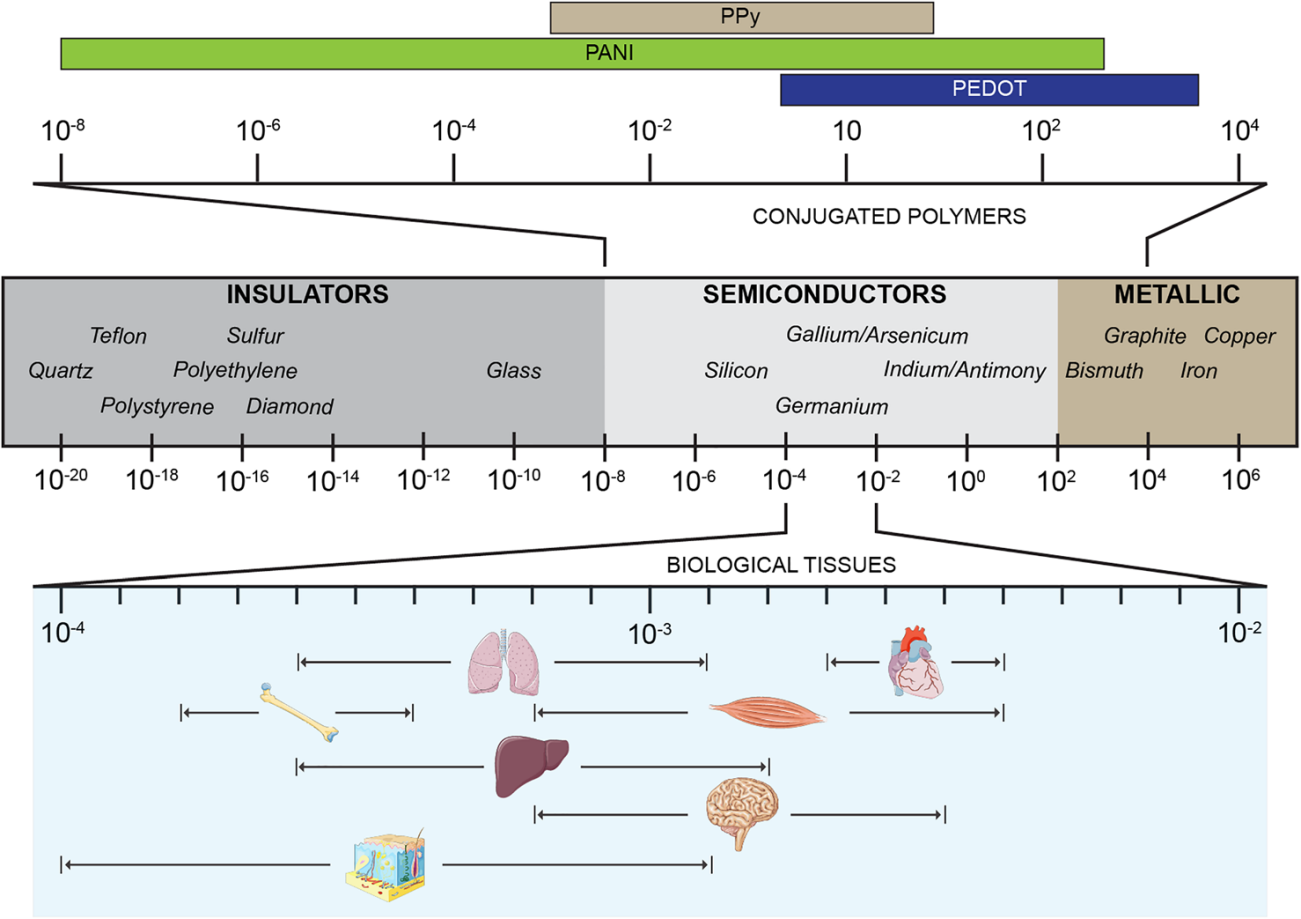
Schematic of typical electrical conductivity values of common materials categorized as insulators, semiconductors, and metals. Top: characteristic conductivities of conjugated polymers. Bottom: average conductivities for biological organs. Values are expressed in S/cm.

Intrinsically conductive polymers (ICPs) have been widely studied in the last 40 years in several engineering fields, since their discovery and development by Heeger, MacDiarmid, and Shirakawa, for which they were awarded the Nobel Prize in Chemistry “for their discovery and development of conducting polymers” in 2000. In 1977, these authors successfully doped polyacetylene which began this era of conductive polymers,^108^ and to understand the importance of this discovery, we here report two citations of Heeger's Nobel lecture, where he described his disclosure as “the fourth generation of polymers” that offers “a unique combination of properties not available from any other known materials.”^109^ The basis of intrinsically conductive polymers is their configuration as conjugated polymers in that, different from common saturated polymers, present the formation of a *π* system created by the electrons of unoccupied *p* orbitals.^109^ In their pristine state, conjugated polymers characteristically possess low conductivity, and to compensate this lacking, external charges are introduced via doping processes such as electrochemical treatments or chemical reactions of oxidation or reduction (chemical doping). ICPs have since then widely used in many fields of engineering and technology. Specifically, in the field of biomedical engineering, polyaniline (PANI), polypyrrole (PPy), and polythiophene have been shown to possess adequate biocompatibility, as well as achieve electrical conductivity values to match those of biological tissues (Fig. 1),^42,43,110,111^ therefore becoming promising materials for biomedical applications.^112^

#### 1. Polyaniline

The first reports on the use of conductive polymers were more than 150 years ago with the work of Letheby^113^ who electropolymerized aniline upon platinum electrodes although the material did not possess the conductivity properties for which it is known nowadays. Thereafter in 1967, stable electronic conductivity in completely dried samples of emeraldine^114^ was established, and since then, interest in PANI has dramatically risen due to the low cost of its monomer and a high yield polymerization reaction.^115,116^ Aniline can be found in three main oxidation states that can be converted from one to the other: leucoemeraldine (pale and reduced), emeraldine either insulator base or conductive salt (green and half-oxidized), and pernigraniline (black and oxidized).^116,117^ The nonconductive emeraldine base can be easily doped via electrochemical or chemical oxidation that alters the number of electrons in *p* orbitals or via protonation, a unique mechanism typical of PANI, whereby the introduction of H^+^ in the molecular chain results in spin-unpairing and a new charge state without changing the total number of electrons.^115,117^ Because of its ease of processing and stability, PANI is often processed in its emeraldine base state and rendered conductive afterwards^115^ via the use of various oxidative agents to switch between reduced/oxidized states.^117^

With its ease of processing and the biological applicability of its reduction/oxidation transitions, PANI has been widely investigated in the biomedical field and in tissue engineering as an electroconductive 2D surface and 3D electroactive scaffold.^118^ However, concerns on the use of PANI in biological applications are related to the lack of biodegradability that can induce chronic inflammation in long-term implants.^119^ A second limitation consists in the potential toxicity caused by the use of solvents for the processing and chemicals—as strong acids—for doping.^120^ Despite these drawbacks, PANI has been widely investigated and huge potential has been shown across the decades, with efforts to increase its biocompatibility via blending with biodegradable polymers and reducing the presence of harmful substances.^119^

#### 2. Polypyrrole

Polypyrrole (PPy) was the first polymer to manifest conductive properties,^121^ with the characteristic conductivity of 7.54 S/cm for “pyrrole black,” the first conductive form of pyrrole achieved via chemical oxidation.^122^ Its conductivity is dependent on many reaction factors and on the choice of the preparation technique, with variable conductivities reported ranging from 0.07 S/cm (Ref. 123) to 90 S/cm (Ref. 124) with the addition of poly(ethylene glycol) during polymerization.^124^ Although widely accepted to be hydrophobic,^125^ unfunctionalized PPy also exhibits hygroscopic characteristics; therefore, it is important to maintain in dry conditions, which limits its biological applications, especially in the physiological environment.^124^

PPy is probably the most investigated conjugated polymer for tissue engineering applications. Being the first polymer to show electrical properties applicable to the technological industry and exhibiting improved conductivity than PANI, this material has been shown to partially replicate the electrical features of metals but possesses a more optimal mechanical match with native biological tissues;^126^ its first application in tissue engineering being 25 years ago.^127^ Despite its wide use in tissue engineering applications, PPy has exhibited cytotoxic effects with reduced cell proliferation when used at high concentrations (30% PPy mixed with polycaprolactone (PCL) and gelatin).^125^ PPy does not degrade in physiological conditions and many efforts to produce a biodegradable mixture via blending with natural polymers have been attempted; however, it has been recommended to maintain the lowest amount possible for *in vivo* applications.^128^

#### 3. Polythiophene

At the time of poly(3,4-ethylendioxythiophene) (PEDOT) discovery—the most investigated compound of the poly(thiophene) family—conductive polymers such as PANI and PPy possessed inadequate conductive stability when placed in contact with oxygen or water, which posed a key limitation for many technological fields.^110^ PEDOT exhibits very unique and specific features, being stable at very high temperatures and humidity and solubility in water when combined with an appropriate counterion and primary dopant, such as poly(styrene sulfonate) (PSS).^110^ PEDOT:PSS transduces charge by both ion and electron/hole exchange, and because of its chemical stability and processability,^129^ it has been the subject of extensive research in the fields of microelectronics,^130^ sensor technology,^131^ and actuation^132^ and has been explored extensively in biological scaffold development, neural implant,^133^ and optoelectronic applications.^134^

Despite PSS being the most utilized counterion and primary dopant with PEDOT, the presence of PSS in excess has its drawbacks from both conductive and biocompatibility perspectives. Moreover, one must take into consideration the crucial importance of the cross-linking treatment to adopt with PEDOT:PSS as this factor is responsible for drastic changes in conductivity.^135^ Indeed, conductivity values for PEDOT:PSS films have been reported in a broad range spanning from 0.2 S/cm up to 4380 S/cm achieved via acetone treatment^136^ or crystallization with sulfuric acid, respectively.^137^ Many effects of the material composition and its processing have been observed on cellular responses.^138^ The so-called “spongelike” capacity of PEDOT:PSS to change the surrounding environment depending on its redox-oxidized state has been shown to have significant effects on the adhesion and proliferation T98G, a glioblastoma multiforme cell line.^139^

To date, little or nothing is known on the potential immune reaction when implanted *in vivo*. Studies on the biological use of PEDOT:PSS have observed a certain level of cytotoxicity when at a particular threshold, for example, a GelMA-based photocrosslinkable hydrogel with PEDOT:PSS at a concentration of 0.3% w/v exhibiting cytotoxicity with C2C12 cells. Researchers have speculated that this toxic effect may be due to the excess of PSS and the subsequent increase in the anionic presence in the environment.^140^ The complete removal of this excess of PSS or the use of a different type of PEDOT would be mandatory for future *in vivo* applications.

## IV. THE APPLICATION OF ELECTROCONDUCTIVE BIOMATERIALS AND ELECTRICAL STIMULATION TOWARDS CARDIAC REGENERATION

### A. The impact of electrical signaling during *in vitro* cardiomyogenesis

Recent decades have seen the development of disruptive advanced manufacturing techniques such as 3D printing, cell reprogramming, and genome editing,^141^ which has fostered a field of research to engineer organoids and organs on a dish.^16^ Despite bounding efforts and advances, current models can often be considered lacking, with shortcomings in fully differentiated and mature cardiac phenotypes, arrhythmias, and reduced strength compared to native tissue.^142,143^ Platforms produced using electroconductive biomaterials could recapitulate the physiological cardiac microenvironment and therefore progress the field of organoid cultures becoming a potent asset. Such an asset could allow one to model the physiological and pathological myocardium toward the study of new drugs and for the study of cellular biology.

#### 1. Delivery of external electrical stimulation

**FIG. 2.**
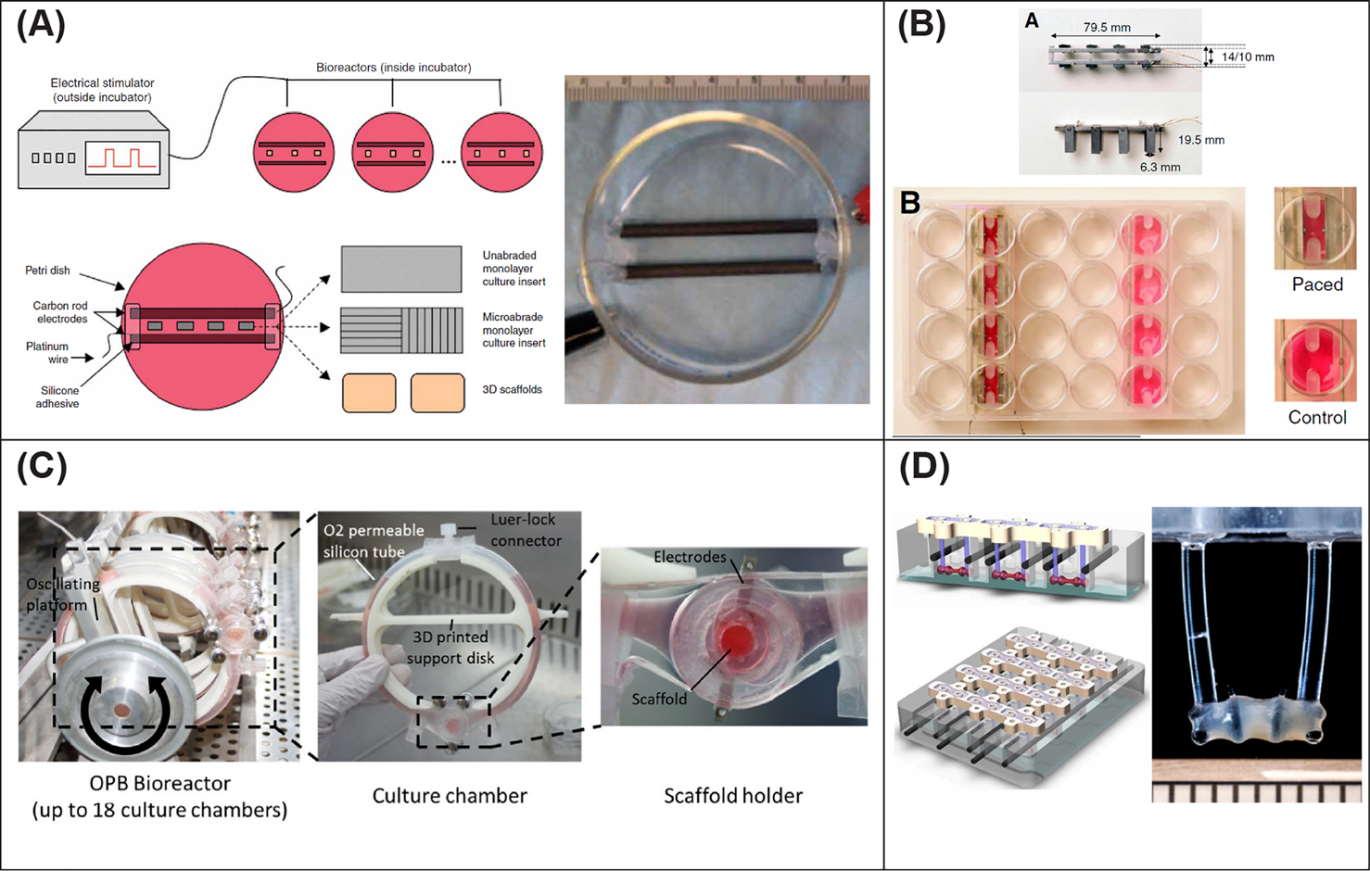
Representative examples of different designs for electrical stimulation bioreactors. (a) Bioreactor consisting of two parallel carbon rods, activated by a Grass stimulator and with the possibility to deliver excitation to both cell monolayers and 3D scaffolds. Reproduced with permission from Tandon *et al.*, “Electrical stimulation systems for cardiac tissue engineering,” Nat. Protoc. 4(2), 155–173 (2009). Copyright 2009 Nature Publishing Group. (b) Bioreactor that can be combined to a standard tissue culture well plate and that can accommodate both paced and unpaced samples. Reproduced with permission from Hirt *et al.*, J. Mol. Cell. Cardiol. 74, 151–61 (2014). Copyright 2014 Creative Commons Attribution (CC BY) license. (c) A dual perfusion-electrical stimulation system that can operate up to 18 systems in parallel and enables monitoring during contraction tests. Visone *et al.*, Sci. Rep. 8, 16944 (2018). Copyright 2018 Creative Commons Attribution (CC BY) license. (d) Evolution of the systems in (a) and (b), where the tissue is held between two flexible pillars. Reproduced with permission from Ronaldson-Bouchard *et al.*, “Advanced maturation of human cardiac tissue grown from pluripotent stem cells,” Nature 556, 7700 (2018). Copyright 2018 Macmillan Publishers Limited, part of Springer Nature.

When modeling physiological stimuli *in vitro* to drive tissue maturation, the application of electrical stimulation had proven to exert a potent influence. Early attempts at three-dimensional *in vitro* cardiac models with electrical stimulation to achieve myocardium maturation are dated to the late 1990s.^144–146^ Since then, great progress in the field has been achieved. In one such instance, neonatal rat ventricular myocytes cultured in ultrafoam collagen sponges had significant improvements in the cell tissue morphology when exposed to 5 days of electrical pacing consisting in 2 ms rectangular pulses at 1 Hz and an intensity of 5 V/cm.^147^ Notably, electrically stimulated groups possessed a decreased nucleic volume, increased mitochondrial number, and more mature sarcomere structure when compared with nonstimulated controls. Pharmacological inhibition of influxing Ca^2+^ exerted only temporary effects which were reversible when constructs were stimulated during cultivation. Since this work, the application of electrical stimulation has become an attractive and effective method to increase CM maturation. Electrically paced cells tend to align in clusters along the direction of the applied electric field lines, an alignment that can be enhanced when combined with substrates with oriented topography.^32,148^ Such an alignment is hypothesized to stem from myoblast mechanotransduction, in that alignment is dictated by a Ca^2+^-independent mediator downstream of the PI3K pathway, a known key regulation factor for both cell-cell fusion during myogenic differentiation and cytoskeleton remodeling. Moreover, myofibrils achieved using this process have developed higher contraction force when they are conditioned with electrical stimulation^149^ [Fig. 2(b)].

Cardiomyocyte pacing has been achieved with bioreactors of differing variations, some of which are illustrated in Fig. 2 such as the conceptual direct model of Tandon *et al.*^150^ [Fig. 2(a)] and the more recent one designed by Visone *et al.*^151^ [Fig. 2(c)] which can both perfuse and deliver electrical stimulation up to 18 independent constructs, while direct observation and monitoring of the tissue function can be performed during contractility tests. Although not the primary focus of this manuscript, one must also acknowledge the influence of mechanical stimulation on cell and tissue fate. An intimate interplay between substrate topography and electrical pacing does exist.^152^ Furthermore, electrical conditioning, in combination with a 125% static tissue straining induced by an external force, has been shown to enhance CM density, size, and alignment of both myofibril and collagen fibers when compared to unstressed tissue, eventually conferring improved contractile strength to constructs.^153^ Moreover, attempts have been made to deploy not only static but also dynamic physical stimulations and achieve an electromechanically active environment similar to the native one.^154^ Studies have brought to light the importance of the relative timing between the two cues, specifically showing improved functional properties when the electrical impulses were delivered at the end of the mechanical stretches.^154^

#### 2. Electroconductive biomaterials as scaffolds

Aiming to mimic the bioconductance of native cardiac tissues, such as the Purkinje fiber network, researchers have investigated the influence of electroconductive biomaterial scaffolds *in vitro*. Electroconductive substrates may present as smarter platforms to direct current flow, synchronize cell beating, and enhance myocardiallike tissue maturation with an increased expression of cardiac markers.^35^

Initial attempts have utilized the addition of metallic components or carbon-based particles to develop electrically conductive scaffolds in cardiac applications. Gold nanoparticles have been combined with hydrogels of hydroxyethyl methacrylate (HEMA)^155^ and with thermosensitive chitosan-based hydrogels.^156^ Both formulations had improved conductivity and upregulated Cx43 expression in CMs^155^ and the expression of cardiac markers in mesenchymal stem cells (MSCs).^154^ In recent years, graphene has garnered attention and been used in combination with both synthetic and biologic polymers. The group of O'Brien^67^ cultured murine embryonic stem cell-CMs on a combined pristine graphene and collagen type I substrates, reporting an increase in cell alignment and CM maturation after electrical stimulation. 3D porous foamlike scaffolds achieved via lyophilization and based both on collagen type I^157^ and GelMA^158^ have been functionalized with rGO and shown to yield increased CM maturation *in vitro* and vasculogenesis when implanted subcutaneously.^158^ Chemical vapor deposition of graphene onto polyethylene glycol (PEG) substrates via a multistep processing involved two intermediate steps with copper foil and poly(methyl methacrylate); Smith *et al.*^159^ achieved oriented micropatterning to mimic the anisotropic conductivity of the native myocardium. The hydrophilicity of the graphene substrate led to significant improvements in cell attachment, sarcomere length, and adult cardiac marker expression. Additionally, it was extrapolated that graphene promoted recycling of Ca^2+^ to the lumen of the sarcoplasmic reticulum in cultured cardiac cells due to an increased intensity of Ca^2+^ transient and upregulation of SERCA2 expression. Other carbon-based materials such as CNTs have imbibed increased strength and conductivity to blended materials and owing to their morphology present nanotopographic cues to cells. When dispersed in culture medium, CNTs exhibit cytocompatibility up to a concentration of 0.032 mg/ml (Ref. 160) and enhance the differentiation of MSCs toward a cardiac lineage when combined with electrical stimulation.^161^ CNTs have been combined with various materials such as within GelMA hydrogels,^35^ within with an elastic polyester polymer [poly(octamethylene maleate (anhydride) 1,2,4-butanetricarboxylate, 124 polymer]^162^ and chitosan-based blend porous scaffolds,^163^ and as a core for coaxial fibers in poly(ethylene glycol)-poly(D,L-lactide) copolymers.^164^

The application of intrinsically conductive polymers is a more recent development in cardiac tissue engineering. PANI has been blended with poly(lactide-co-glycolide) (PLGA) and processed via electrospinning to achieve aligned conductive fibrous meshes, with CMs seeded on this substrate grouping in isolated clusters with Cx43 expression and synchronous beating, which can be influenced when a pacing regime is applied.^152^ Similar beneficial effects have been observed using H9C2, a rat cardiac myoblast cell line, which when seeded on a thin film of conductive polylactic acid (PLA)-aniline pentamer and paced had increased cell attachment, spreading, and proliferation as well as increased levels of intracellular calcium with developed “pseudopodia,” deemed to be precursors of myocardial intercalated disks.^165^ PPy has been combined with PCL in both 2D films^166^ and 3D electrospun fibrous scaffolds,^167^ and its effect investigated on mouse atrial myocytes cell line HL-1 and primary rabbit CMs, respectively. The presence of PPy promoted increased Ca^2+^ propagation velocity and decreased calcium transient durations in 2D, enhanced cellular alignment in 3D, while Cx43 expression was significantly upregulated in both studies.

Electroconductive biomaterials have also improved CM maturation in the absence of electrical stimulation. In the study of Wu *et al.*,^168^ CNT-based biomaterials alone have been shown to increase the expression of cardiac features and markers. A conductive blend termed “Yarns,” composed of PCL, silk fibroin, and CNTs, was processed using a wet–dry electrospinning process which was then combined with GelMA. To model the multioriented architecture of native myocardium, two orthogonal layers of aligned CMs cocultured with a third layer of endothelial cells were constructed.^168^ The application of the electroconductive scaffold enhancing cellular function in the absence of electrical stimulation has also been demonstrated using an aniline-derivative polyurethane, whereby an increase in neonatal rat CMs Troponin T Type 2 and Actinin alpha 4 gene expression was observed when compared to a nonconductive PCL control after 3 days.^169^

#### 3. Maturation of human induced pluripotent stem cells

Human induced pluripotent stem cells (hiPSCs) have led the attention of the scientific community^170^ with encouraging progress in the field of cardiac regeneration, having repopulated a decellularized mouse heart,^171^ and demonstrated success in regenerating an infarcted primate heart.^27^ Their use provides an easily available solution without moral dilemma; however, they have drawbacks as incomplete terminal differentiation poses a risk of teratoma *in situ.*^172^ Despite the efforts of many research groups, complete differentiation to adult cardiac phenotypes remains inadequate and it varies according to the batch of cells being used.^173^

Currently, electroconductive polymers are been applied to enhance the maturation of hiPS-CMs *in vitro*. Studies have developed electromechanically active fibrous electrospun PLGA scaffolds functionalized by electropolymerization deposition of PPy,^174^ which had the capability to contract due influxing of ions from the surrounding media into the PPy coating when an electrical pacing was applied, therefore working as actuators. Cardiomyocyte differentiation of hiPSCs seeded on these actuating platforms had increased Actinin, NKX2.5, GATA4, and Myh6 expression when compared to a noncoated PLGA and unstimulated PPy/PLGA scaffolds. In a separate study, electrospinning of a blend of PANI and polyethersulfone has been shown to yield differentiation of cardiovascular disease specific iPSCs toward a cardiac phenotype with an upregulation of NKX2.5, GATA4, NPPA, and TNNT2.^175^ Despite toxicity reported at high concentrations, PEDOT:PSS at 0.26 w/w% has been incorporated within biohybrid hydrogels of both collagen type I and alginate.^176^ The presence of PEDOT:PSS in these hydrogels enhanced the maturation of rat primary CMs and hiPSC-CMs *in vitro* with faster and wider contraction, as well as increased sarcomeric length comparable with CM adult values after 11 days of culture and electrical stimulation.

Conditioning via electrical stimulation has accelerated the differentiation of hiPS-CMs *in vitro* with an efficacy of 80%, compared to 60% with no stimulation; moreover, once implanted in an *in vivo* MI mouse model, the group conditioned with electrical pacing yielded a reduced infarct region, increased ejection fraction, and left ventricle fractional shortening. However, the risk of arrhythmia and the presence of a heterogenous CM population were highlighted as limiting challenges of the study.^177^

### B. Cardiac patches

Mechanical support of the infarcted myocardium is a tried approach to restrict adverse ventricular dilatation by application of cardiac patches on the external surface of the myocardium.^178^ Research in the field has focused on the development of biomaterial meshes with suitable ranges of elasticity for optimal mechanical support, often incorporating cell therapies or growth factors,^179^ and often designed to allow a minimally invasive *in situ* delivery.^28^ Although mechanically sufficient, this approach does not address dysfunctional electromechanical coupling due to scar tissue. Electroconductive biomaterials have been adopted to manufacture patches that not only mechanically support the ventricle but also can potentially bridge the electrical propagation across the nonconductive infarcted area, aiming to achieve a restoration of the native conduction system.^35^

**FIG. 3.**
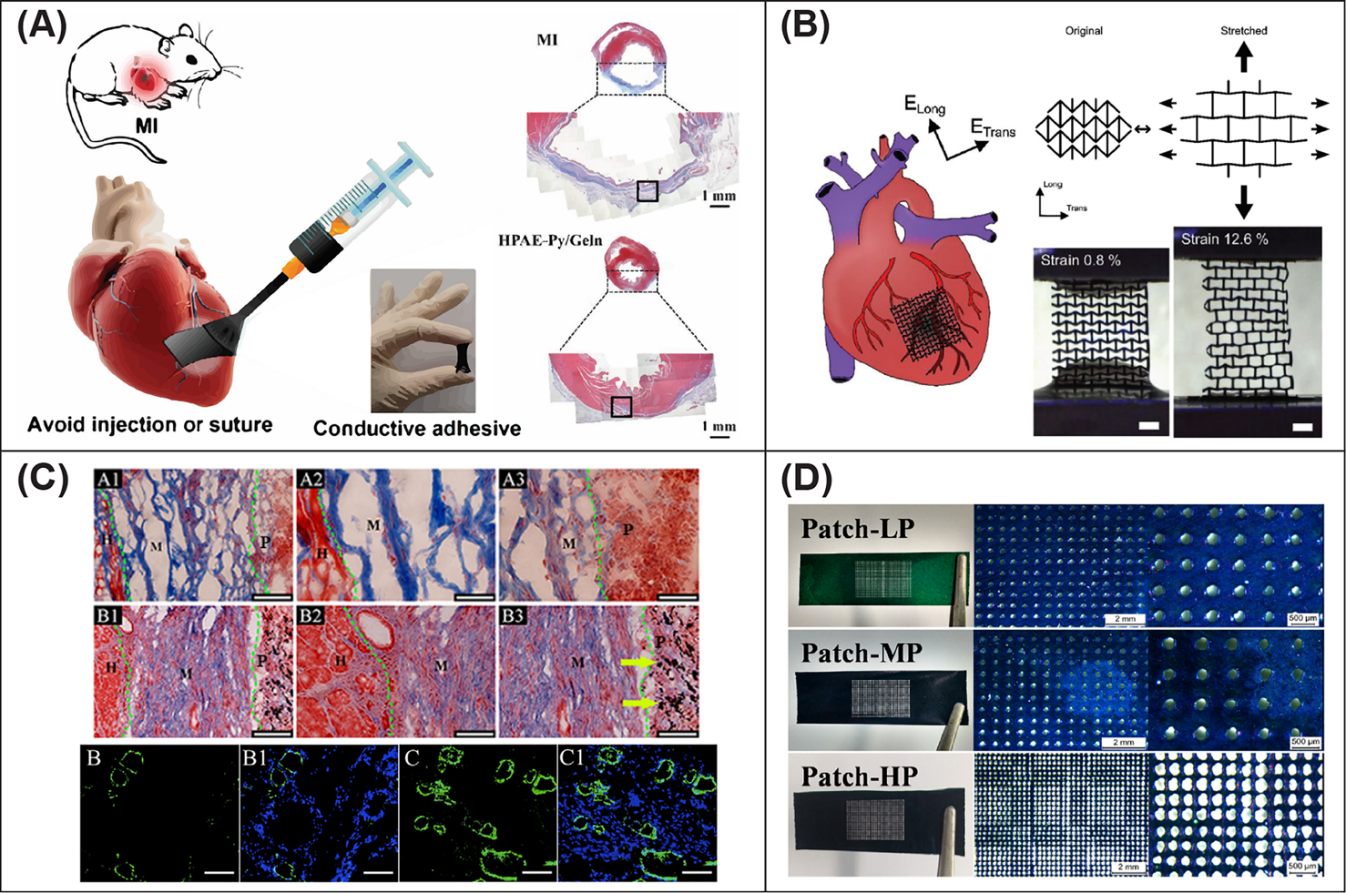
Smart electroactive cardiac patch designs. (a) A paintable hydrogel-patch that can be directly applied to the myocardium and drive tissue recovery. Reproduced with permission from Liang *et al.*, “Paintable and rapidly bondable conductive hydrogels as therapeutic cardiac patches,” Adv. Mater. **30**, 1704235 (2018). Copyright 2018 Wiley-VCH Verlag GmbH & Co. KGaA, Weinheim. (b) A conductive patch with auxetic design that can recapitulate the anisotropy characteristic of the myocardium. Reproduced with permission from Kapnisi *et al.*, Adv. Funct. Mater. 28, 1800618 (2018). Copyright 2018 Creative Commons Attribution (CC BY) license. (c) Preseeding of CMs on a chitosan-PPy patch promoted engraftment and cardiac function improvement. Reproduced with permission from Song *et al.*, Appl. Mater. Today 15, 87–89 (2019). Copyright 2019 Creative Commons Attribution (CC BY) license. (d) Chitosan-PANI film with porosity controlled via laser ablation and processed with three different pore sizes. Reproduced with permission from Hoang *et al.*, “Porous and sutureless bioelectronic patch with retained electronic properties under cyclic stretching,” Appl. Mater. Today 15, 315–322 (2019). Copyright 2019 Elsevier Ltd.

Conductive cardiac patches have been fabricated by electrospinning blends of PCL:PANI nanofibers^180^ and fabricating alginate scaffolds doped with gold nanowires.^181^ Both these approaches established an advantage of using conductive substrates to improve the cell response. The first study reports a significant role in driving human MSCs differentiated into CMs,^180^ while the second demonstrated a bridging of electrical coupling between adjacent primary rat CMs and fibroblasts, with higher cardiac marker expression and more synchronous beating than when using pure alginate.^181^ GelMA patches doped with CNTs have demonstrated a significant enhancement of *in vitro* electrical functionality when compared with nonconductive control patches, namely, the rhythmic contractility of cell seeded patches could assume a tubular shape when floating in medium which could be controlled when electrically stimulated.^35^ Subsets of conductive particles and polymers can be processed via several techniques to achieve cardiac patches, such as incorporation of CNTs in electrospun PCL,^182^ 3D bioprinting of alginate or methacrylated collagen hydrogel meshes crosslinked and reinforced with CNTs,^183^ and laser ablation to micropattern chitosan films which can be subsequently functionalized with PANI via *in situ* polymerization [Fig. 3(d)].^184^ Despite their relevance in the field, the available reports of these works are currently limited to *in vitro* evaluation.

More recently, the application of conductive cardiac patches *in vivo* has been reported, with many solutions describing chitosan as a base biomaterial for the incorporation of electroconductive fillers. The application of these patches *in vivo* improved the conduction propagation in infarcted hearts when PANI,^185,186^ PPy,^26^ graphene oxide, and gold nanosheets^187^ were adopted as conductive fillers. When preseeded with CMs^188^ [Fig. 3(c)] and hiPSC-CMs^187^ prior to implantation, graphene oxide and gold nanosheets have demonstrated an overall improvement in cardiac function after 4 and 5 weeks, respectively. An approach to recapitulate the mechanical and electrical anisotropy of the native human myocardium has utilized excimer laser microablation on chitosan films to generate an auxetic design, later functionalized to be conductive via deposition-coating of PANI [Fig. 3(b)]. Although promising *in vitro*, modest effects were observed *in vivo* with no increase in conduction velocity of the electrical beating impulse compared to a mesh with standard design that was previously fabricated by the same research group.^185^ Another study evaluated a paintable adhesive hydrogel-patch based on a dopamine-PPy blend. This material could be directly applied directly on the heart without sutures and was reported to promote cardiac function recovery and revascularization of the infarcted myocardium [Fig. 3(a)].^189^ A final mention is the development of a two-layer hybrid construct based on a flexible collagen type I hydrogel that conferred mechanical support and consisted of a matrix of fibrous collagen doped with gold nanoparticles providing electrical properties. This platform induced upregulation of Cx43 expression *in vitro* after electrical stimulation, while it improved cardiac function and vasculogenesis at 5 weeks *in vivo*, without provoking proinflammatory differentiation of macrophages.^190^

### C. Injectable hydrogels

In order to alleviate the loss of myocardial volume following MI, together with facilitating a minimally invasive approach, injectable hydrogels have been adopted to not only restore healthy heart geometry but more importantly to locally deliver cell-based treatments^143^ with or without other functional therapeutics.^191^ As electroconductive biomaterials have shown promise on cell behavior *in vitro*, several groups have investigated the synthesis of electrically conductive hydrogels to recover cardiac function.

**FIG. 4.**
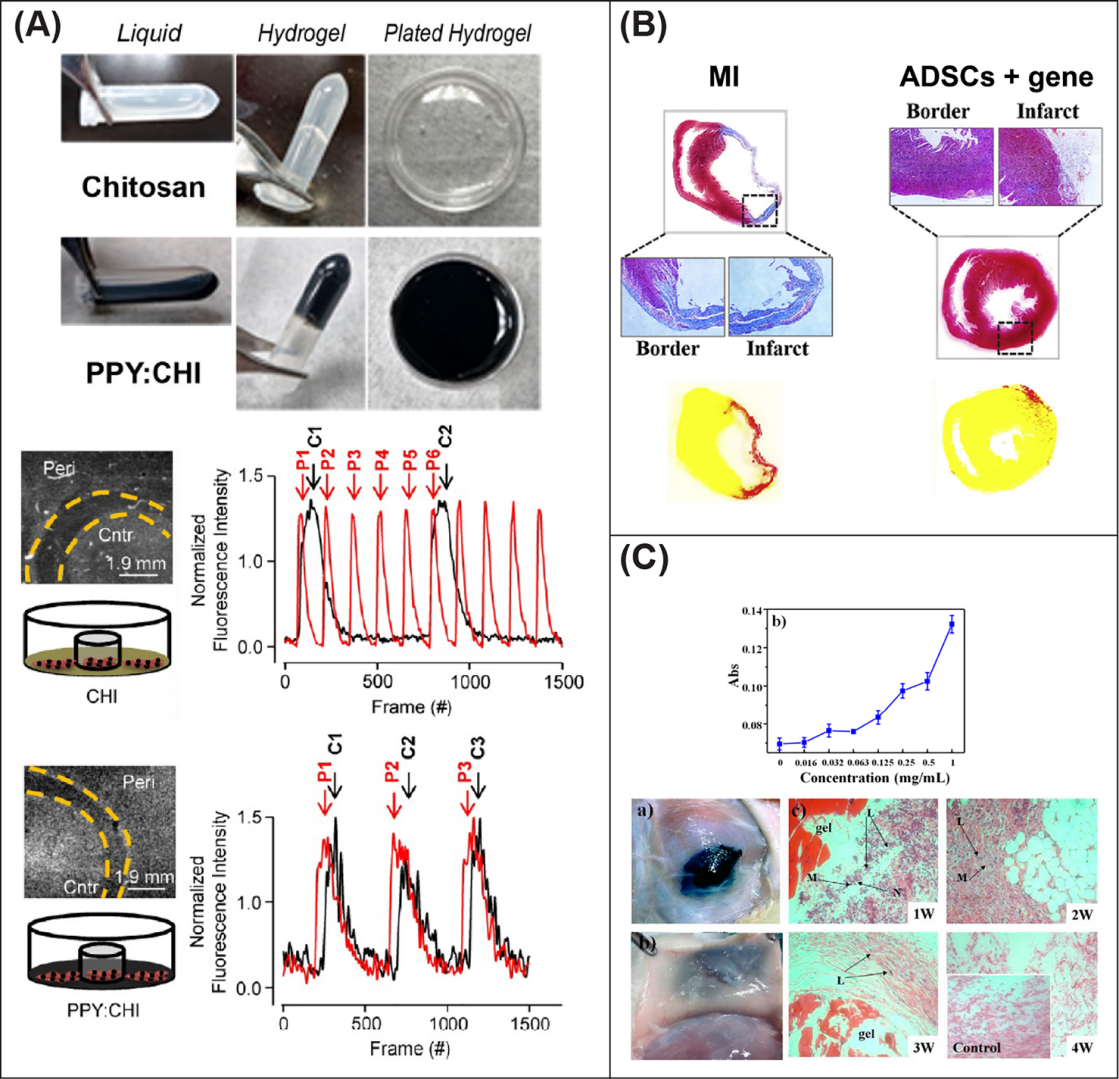
Promising injectable conductive hydrogels for *in situ* myocardium regeneration. (a) A chitosan-PPY hydrogel can electrically couple two separated CM populations. Reproduced with permission from Cui *et al.*, Theranostics **8**(10), 2752 (2018). Copyright 2018 Creative Commons Attribution (CC BY) license. (b) Adipose derived stem cells and pDNA incorporated in a conductive hydrogel, showed to improve the functionality of the heart *in vivo*. Reproduced with permission from Wang *et al.*, “An injectable conductive hydrogel encapsulating plasmid DNA-eNOs and ADSCs for treating myocardial infarction,” Biomaterials 160, 69–81 (2018). Copyright 2018 Elsevier Ltd. (c) A PANI-doped hydrogel showed antioxidant effects after subcutaneous implantation. Reproduced with permission from Cui *et al.*, “In vitro study of electroactive tetra-aniline-containing thermosensitive hydrogels for cardiac tissue engineering,” Biomacromolecules 15, 1115–1123 (2014). Copyright 2014 American Chemical Society.

Chitosan has been adopted to create hydrogels for cardiac repair in addition to the *in vitro* applications and cardiac patches discussed previously. Combining chitosan with PPy to fabricate a hydrogel has facilitated electrical coupling in skeletal muscle tissue *ex vivo*^192^ and between isolated CM populations *in vitro*^193^ [Fig. 4(a)]. *In vivo* implantation of chitosan/PPy hydrogels in an infarcted rat model has improved electrical impulse propagation across scarred tissue, decreased the QRS interval with an increase in conduction velocity, and enhanced the cardiac function when compared to a nonconductive hydrogel of a similar nature.^192,193^ An oxidized dextran crosslinked chitosan-graft-polyaniline hydrogel has also been used to develop electroresponsive smart drug carriers loaded with amoxicillin and ibuprofen and for antibacterial treatments. This hydrogel was pH-responsive and exhibited good biocompatibility both *in vitro* on L929, a mouse fibroblast cell line, and *in vivo* via subcutaneous implantation in a rat model with almost total resorption at 28 days.^191^

Among the first reports demonstrating the efficacy of conductive nanomaterials in heart regeneration, a gelatin-based hydrogel doped with SWCNTs has yielded an enhanced expression of cTnT and Cx43 in neonatal rat CMs *in vitro*, with synchronous beating after 8 days of culture. The application of this material with a cargo of primary rat CMs to a rat model of MI found host vasculature invading the hydrogel after 1 week, while at 4 weeks, evidence that cells and scaffolds partially migrated into the host myocardium was observed. However, the conductive hydrogel had the highest amount of M1 macrophages at the interface. The application of this conductive hydrogel led to a series of improvements in the heart function, such as an increased fractional shortening and ejection fraction and reduced progression of left ventricle enlargement. Molecular mechanisms triggered by the presence of SWNTs were investigated with speculation that the beneficial effects on cardiac repair can be related to the integrin-mediated mechanotransduction pathway, specifically of integrin-linked kinase (ILK), protein kinase B (AKT), and β-catenin.^194^ However, the conductive hydrogel had the highest amount of M1 macrophages at the interface between the host tissue and hydrogel when compared to the nonconductive control. Strategies encapsulating plasmid DNA (pDNA) into biomaterials are an alternative approach to enhancing stem cell differentiation *in situ.*^18^ Myocardial delivery through a 22-gauge needle of a conductive hydrogel composed of graphene oxide, GelMA, and pDNA encoding VEGF_165_ (Ref. 29) was able to induce neoangiogenesis in a paracrine manner, without cytotoxic effects. Similar positive outcomes in terms of tissue healing and angiogenesis have been achieved also via the implantation of a tetraaniline/hyaluronic acid conductive hydrogel delivering pDNA encoding endothelial nitric oxide synthase (eNOS) and adipose derived stem cells [Fig. 4(b)].^25^ Improvement in the heart function at 4 weeks has been achieved by the administration of adipose tissue-derived stromal cells encapsulated in a PEG diacrylate melamine crosslinked with thiol-modified hyaluronic acid and doped with graphene oxide in a rat model of MI.^195^

CNTs^35^ and PANI^196^ have revealed intrinsic radical scavenging activity that could be a key factor to modulate regeneration of the heart as reactive oxygen species are typical hallmark of the ischemic myocardium.^197^ *In vitro* evaluation at 7 days on H9C2 cells demonstrated that a tetra-aniline copolymer P(NIPAM-mPEGMA-MDO-MATA) (PN-TA) may reduce the free radical-mediated oxidative cardiac damage; moreover, the application of electrical stimulation enhanced the cellular response material biocompatibility [Fig. 4(c)]. To evaluate the antioxidant effect *in vitro*, the authors used 2,2-diphenyl-1-picrylhydrazyl (DPPH) as a model, showing how the introduction of the antioxidant material improved significantly cell viability.^196^ The authors repeated similar experiments adopting a different material^198^ mixing tetraaniline copolymers and cyclodextrin. Again, histological staining of subcutaneous implantation demonstrated biocompatible response with almost no inflammatory response and sensible reduction of fibroblastic capsule at 3 weeks.

## V. THE FUTURE OF ELECTROCONDUCTIVE BIOMATERIALS IN TISSUE ENGINEERING AND THEIR BEHAVIOR IN LONG TERM SETTINGS

It is becoming increasingly evident that electroconductive biomaterials will pose a significant factor in tissue engineering in the coming years to achieve smart solutions in the field of not only cardiac tissue engineering but also other aetiologies of disease. Based on the abundant evidence discussed in this review, electroconductive biomaterials and electrical stimulation are critical factors to be considered in achieving success in the maturation of cardiac organoids and to provide auxiliary paths for the conduction of action potentials within the impaired myocardium. In terms of CM differentiation, improvements have been reported with electroconductive biomaterials alone without the presence of electrical stimulation and vice versa when electrical stimulation was applied in the absence of an electroconductive biomaterial. As discussed above, however, when applying these two features simultaneously, even increased success is obtained, highlighting the importance of applying these two factors together (Table I).

**TABLE I.** Overview of electroconductive biomaterial systems employed in the field of cardiac tissue engineering and cardiac biomaterials.

	Heart models *in vitro*		Smart cardiac patches *in vivo*		Injection of hydrogels *in vivo*	
Electroconductive biomaterial	Fabrication	Findings	Fabrication	Findings	Fabrication	Findings
CNTs	GelMA hydrogels^35^	Nanotopographic cues to cells ↑ Cardiac markers in MSCs	…	…	Gelatin-based^194^	↑ cTnT and Cx43 *in vitro*, angiogenesis *in vivo*
	Elastic polyester^162^					
	Chitosan-based^163^					
	PEG-poly(D,L-lactide)^164^					
	Wet-dry electrospinning YARNS + GelMA^168^	↑ Cardiac feature and markers w/out electrical stimulation				
Graphene	Film: pristine graphene + collagen^2^	↑ Cell alignment, hES-CM maturation	GO + AuNPs + chitosan^186^	↑ Conduction velocity and contraction	GO with GelMA and pDNA (VEGF_165_)^29^	↑ Angiogenesis
	Lyophilization: rGO + collagen^157^	↑ CM maturation				
	Lyophilization: GelMA^158^	↑ CM maturation			GO with PEG diacrylate and adipose derived stem cells (ADSCs)^195^	↑ α-SMA and Cx43 *in vivo*
	Coating PEG via CVD^159^	↑ cell attachment ↑ sarcomere length				
Metallic NPs	HEMA hydrogels^155^	↑ Cx43 in CMs	Collagen hydrogel + collagen fibers^190^	↑ Heart function, vascularization, absence of proinflammatory response	…	…
	Chitosan hydrogels^156^	↑ Cardiac markers in MSCs				
PANI	Electrospinning in blend with PLGA^152^	↑ Cx43 expression, synchronous beating	Chitosan^232^	↑ Heart function, no induction of arrhythmias	Chitosan as smart drug carriers^191^	Controlled inflammatory response *in vivo*
	Film in PLA^165^	↑ Cell proliferation, development of pseudopodia			Hyaluronic acid, pDNA (eNOS), and ADSCs^25^	↑ Angiogenesis and tissue healing
	Polyurethane^169^	↑ TNNT2 and Actinin alpha 4 gene w/out electrical stimulation	Microablation of the chitosan film^9^	Auxetic design, mechanical and electrical anisotropy	Cyclodextrin^198^	↓ Inflammatory response, fibroblastic capsule *in vivo*
	Electrospinning in blend with polyethersulfone^175^	↑ NKX2.5, GATA4, NPPA, and TNNT2				
PPy	Film in PCL^166^	↑ Ca^2+^ propagation velocity ↑ Cx43	Paintable adhesive dopamine blend^189^	↑ Heart function and vascularization	Chitosan^192^	Electrical coupling *in vitro* and *ex vivo*
	Electrospinning in blend with PCL^167^	↑ Cellular alignment ↑ Cx43	Chitosan gel foam^26^	↑ Conduction velocity absence of arrhythmias		
	Coating on PLGA electrospun scaffold^174^	↑ Actinin, NKX2.5, GATA4, Myh6; actuation ability				
PEDOT	Collagen/alginate hydrogel^176^	↑ Increased sarcomeric length; faster and wider contraction	…	…	…	…

Despite the advances and hype for electroconductive biomaterials in this field, none of the materials investigated in this review satisfy all the requirements for stable and successful *in vivo* applications.

To be a suitable candidate for any of the three categories mentioned in this review—scaffolds for *in vitro* models, cardiac patches, or injectable hydrogels—the biomaterial needs to address many factors. Mimicking physiological bioconductance of different organs is attainable with these materials; however, one must keep in mind that the conductive properties of many of these compounds may diminish in physiological environments. Engrafted material should integrate appropriately with host networks to avoid risk of arrhythmia or worse still and add a pathway that is detrimental to electrophysiological signaling *in vivo*. The compound needs to be processable into useful morphologies, such as defined macroscopic porous architectures and mechanical properties suitable for the *in vitro* or *in vivo* applications, which is especially important considering the anisotropic nature of the myocardium.

An ideal candidate material should not induce any toxic response at cellular or systemic levels and do not induce immune reaction or chronic inflammatory responses; however, the performance and translation of the here presented materials to the clinic have yet to be seen. Indeed, *in vivo* experimentation has been limited to subcutaneous or short-term studies, leaving unsolved many open queries regarding the long-term toxicity of these materials *in vivo* and their interplay with our innate and adaptive immune system.

A tenet of tissue engineering often focused upon is the concept of biodegradable scaffolds providing initial structural support that gradually degrades as the host tissue regenerates. None of the electroconductive materials we have described are known to be metabolized *in vivo*. Aiming to generate conductive degradable biomaterials, some groups have explored the combination of a conductive polymer with a degradable matrix or hydrogel.^29,192^ However, even succeeding in this method, the fate of electroconductive by-products released in the body is not clear as there is no univocal proof of their clearance through standard metabolic pathways.^199,200^ One main concern is the penetration capability of these by-products into surrounding tissues and cells, as it is well established that nanoparticles with diameters less than 40 nm can penetrate both the cell membrane and the nucleus with the risk to generate a broad range of reactions, such as the change in nucleus architecture and size or also affecting affect DNA methylation.^200^ *In vivo* studies have shown contradictory results regarding the toxicity of extrinsically conductive materials, and negative effects have already been described for CNTs,^201^ Graphene,^202^ and NPs^89^ which describe the infiltration of byproducts to internal organs and systemic circulation. However, a lack of univocal standard protocols for toxicity evaluation *in vivo* has led to ambiguity in these findings.^203^ Given their recent development, the *in vivo* evaluation of MXene and ICP is at an early stage. Their use in the field of implantable electronics suggests an acceptable tolerance of these substances when used as coatings^204^ and an overall concentration-dependent toxicity.^205^

The regulatory track to get a new material to the clinical phase that requires a new substance to be accepted—and not only cleared—is a long and expensive process that may discourage their introduction to the market. Indeed, despite metallic NPs being historically the most tested conductive materials, few iron-based nanoparticles have been approved for their use as contrast enhancement reagents for medical imaging and no AuNPs have been approved to date yet by the Food and Drug Administration.^206^

Potentially, an inert graft composed of a fully nondegradable material, able to interact with the host without chronic inflammatory reaction or immune response, may be a more suitable solution. In recent years, growing expectations have been raised on the use of PEDOT and its derivatives. Because of its higher stability and conductivity compared to the other intrinsic conductive polymers, this compound may be the most suitable candidate for electroconductive grafts or scaffolds, and it has been shown to be manufactured into three-dimensional structures without the use of a complementary supporting material.^49^ However, as we have been pointed out, long-term toxicity both *in vitro* and *in vivo* is yet to be evaluated. Notably, the presence of PSS as the counterion has shown to lead to the toxic effect, and therefore, a full cleavage of the unreacted leftovers of this molecule must be guaranteed. One strategy to overcome this and increase the biocompatibility and biofunctionality of PEDOT is to incorporate biodopants such as dextran sulfate or alginate, which have been shown to increase the absorption of fibronectin and collagen, respectively.^207^

The growth and application of electroconductive biomaterials are testament to their potential for tissue engineering applications and especially for cardiac regeneration. As *in vitro* models do not require a strict characterization of their degradability and long-term effects of their by-products at a systemic scale, it is most likely that this application will see impact sooner where there is less risk and more control over electrical stimulation. However, with increasing advances in polymer chemistry, greater understanding of degradation kinetics, and the discovery of biological moieties that are used to improve material performance, electroconductive implants in cardiac settings could one day become a routine therapeutic option.
